# Investigating computational models for diagnosis and prognosis of sepsis based on clinical parameters: Opportunities, challenges, and future research directions

**DOI:** 10.1016/j.jointm.2024.04.006

**Published:** 2024-07-10

**Authors:** Jyotirmoy Gupta, Amit Kumar Majumder, Diganta Sengupta, Mahamuda Sultana, Suman Bhattacharya

**Affiliations:** 1 Department of Computer Science and Engineering (IOTCSBT), Future Institute of Technology, Kolkata, West Bengal, India; 2 Department of Electronics and Communications Engineering, Future Institute of Technology, Kolkata, West Bengal, India; 3 Department of Computer Science and Engineering, Heritage Institute of Technology, Kolkata, West Bengal, India; 4 Department of Computer Science and Engineering, Guru Nanak Institute of Technology, Kolkata, West Bengal, India

**Keywords:** Sepsis, Computing methodologies, Early prediction of sepsis, Mortality prediction of sepsis, Machine learning, Artificial intelligence

## Abstract

This study investigates the use of computational frameworks for sepsis. We consider two dimensions for investigation – early diagnosis of sepsis (EDS) and mortality prediction rate for sepsis patients (MPS). We concentrate on the clinical parameters on which sepsis diagnosis and prognosis are currently done, including customized treatment plans based on historical data of the patient. We identify the most notable literature that uses computational models to address EDS and MPS based on those clinical parameters. In addition to the review of the computational models built upon the clinical parameters, we also provide details regarding the popular publicly available data sources. We provide brief reviews for each model in terms of prior art and present an analysis of their results, as claimed by the respective authors. With respect to the use of machine learning models, we have provided avenues for model analysis in terms of model selection, model validation, model interpretation, and model comparison. We further present the challenges and limitations of the use of computational models, providing future research directions. This study intends to serve as a benchmark for first-hand impressions on the use of computational models for EDS and MPS of sepsis, along with the details regarding which model has been the most promising to date. We have provided details regarding all the ML models that have been used to date for EDS and MPS of sepsis.

## Introduction

Sepsis may lead to end-stage organ dysfunction and even death. Prognosis is highly dependent on early diagnosis and the subsequent treatment. “Septic shock” is the critical state of the sepsis patient when blood pressure falls detrimentally, and vital organs start getting lesser oxygen.^[^^1^^]^ This medical emergency can become fatal at any age especially affecting those with chronic health conditions. Mathematical modeling of sepsis can provide significant insight of the disease.^[^^2^^,^^3^^]^ The ever-growing influx of laboratory and hospital data along with patient health history allows quantitative computational approaches to investigate correlations between clinical factors and sepsis fallout. Statistical modeling approaches are used for early stage detection of sepsis and hence a reduction in mortality rate. The emerging domain of explainable artificial intelligence (XAI) further enhances these models’ performance with explainability and thus improving trust and transparency of the practitioners.^[^[4], [5], [6], [7], [8], [9], [10]^]^

This study addresses two research statements: (1) investigate computational models designed for the early diagnosis of sepsis (EDS) onset and (2) explore computational models tailored for mortality prediction in intensive care unit (ICU) sepsis patients (MPS).

**Figure 1:**
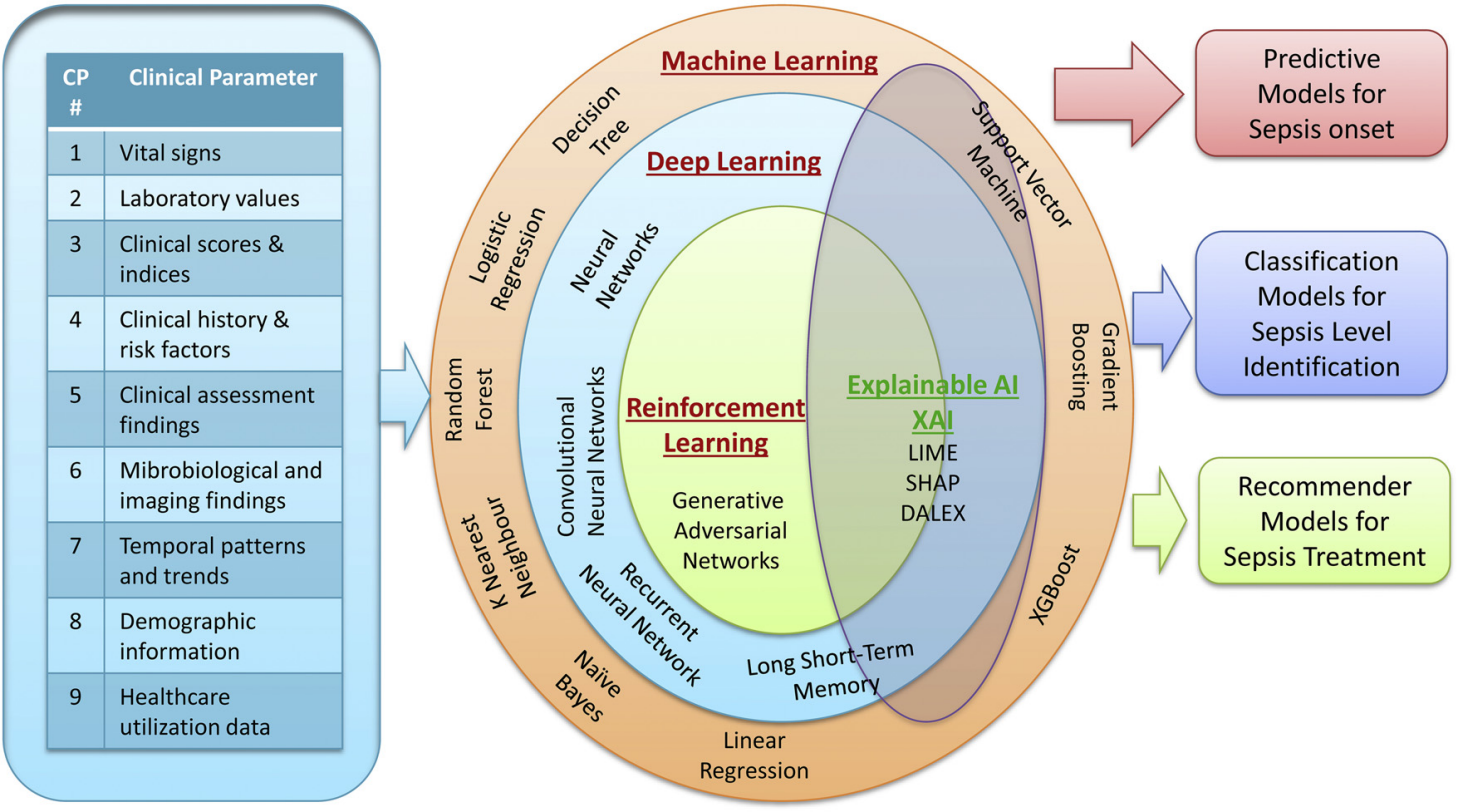
Different dimensions for use of learning models on clinical data. AI: Artificial intelligence; CP: Clinical parameter; DALEX: Descriptive machine learning explanations; LIME: Local interpretable model-agnostic explanations; SHAP: Shapley additive explanations; XAI: Explainable artificial intelligence.

The input features for the computational models have been derived from the feature vector (heart rate, saturation, body temperature, pH, white blood cell [WBC] count, respiratory rate, systolic blood pressure, diastolic blood pressure, bilirubin, pulse oxygen saturation [SpO_2_], glucose, age, bicarbonate, hemoglobin, platelet, shock index, creatinine, lactate, weight, and potassium). The major clinical parameters that serve as the input for the computational models are presented in Figure 1. The various machine learning (ML) models that have been used to analyze those clinical parameters are also provided.

### Existing computational approaches

The existing computational approaches can be divided into four major clusters. Computational models leverage temporal analysis techniques to monitor patient data over time and to identify patterns or anomalies that may indicate the EDS.^[^^11^^,^^12^^]^ Time series analysis, such as sequential pattern mining and recurrent neural networks (RNNs), has been employed to capture dynamic changes in vital signs, laboratory results, and clinical notes. ML regression algorithms, including linear regression, decision tree (DT), gradient boosting (GB), and neural network (NN), have been employed to predict the mortality rates.^[^^13^^,^^14^^]^ Several ML algorithms have been explored, including logistic regression (LR), random forest (RF), support vector machine (SVM), and deep learning (DL) models, to create classification models for EDS.^[^^15^^,^^16^^]^ These models make use of features extracted from patient data, encompassing vital signs, laboratory values, and demographic information utilizing established risk scoring systems such as the sequential organ failure assessment (SOFA) score and acute physiology and chronic health evaluation (APACHE) score.^[^^17^^,^^18^^]^

## Computational Approaches for Prediction, Classification, and Personalized Treatment Plan

We observed three outcomes of ML models based on clinical parameters given in Figure 1. Rello^[^^19^^]^ presented the latest challenges with respect to infectious disease management. O'Reilly et al.^[^^20^^]^ discussed how artificial intelligence (AI) can optimize sepsis management. Yang et al.^[^^21^^]^ reviewed the latest 34 pertinent articles on the data processing abilities of AI and its potential impact on improving EDS and mortality prediction rate for sepsis patients (MPS). Fleuren et al.^[^^22^^]^ pointed out that clinical implementation of individual and ensemble models is currently scarce, even though they tend to outperform biomarkers and traditional scoring criteria such as systemic inflammatory response syndrome (SIRS)/SOFA. Testing the significant 39 covariates (*P* <0.5) successively in a univariate model, followed by a multivariate model, shows that heart rate, respiratory rate, temperature, lab values, and arterial blood gas values contribute significantly towards univariate analysis. However, only temperature, lab values, and model type contributed the most in the multivariate analysis. Further, systematic reporting is necessary for easy data aggregation and reliable interpretation.^[^^22^^]^

### Conventional computational models based on clinical scores

Presently, computational tools such as SIRS, APACHE II, and SOFA, aid the doctors in ICUs. APACHE II investigates clinical parameters and predicts useful analytical solutions to the doctor in the form of a score. The SOFA model focuses on the organ function of a patient to assess whether the natural immune system is registering the treatment or resisting it. According to the SIRS severity spectrum of sepsis, the mildest form shows at least two of the abnormalities, such as temperature >38 °C, heart rate >90 beats/min, respiratory rate >20 breaths/min, and along with WBC >12,000 cells/mm^3^. In uncomplicated cases, a sepsis patient might develop hypotension responsive to infusion of intravenous (IV) fluids along with one or more organ dysfunction. In very severe sepsis cases, the patient experiences hypotension resistant to IV fluids with an elevation of lactate level >2 mmol/L, better known as septic shock. A streamlined version of SOFA, known as quick-SOFA (qSOFA) score, was introduced that can be assessed by physical examination of three criteria, i.e., respiratory status, hemodynamic, and any degree of altered mentation. The conventional models for prediction do not consider the early onset of the disease due to inadequate sensitivity of the models, thereby enhancing probabilities for delayed treatment. As the conventional models primarily rely on clinical parameters, they do not consider the patient's response to the infection or the presence of any specific pathogens for accurate risk stratification due to dependence on physiological parameters.

### Computational approaches for EDS

In computational tools designed for EDS, the significant decline in algorithm performance is primarily attributed to high rates of false alarms and missed detections.^[^^23^^]^ They introduced conformal multidimensional prediction of sepsis risk (COMPOSER), a comprehensive DL model capable of EDS within a clinically relevant timeframe spanning from 4 h to 48 h in advance. The COMPOSER model was crafted to offer local interpretability while maintaining consistent performance. The model reported high area under the curve (AUC) of 0.925–0.953 on the ICU cohort and 0.938–0.945 on the Emergency Department cohort. Moor et al.^[^^24^^]^ developed a DL system for EDS and externally validated the model in a large international, multi-center cohort of about 136,478 ICU patients. With harmonized ICU patient data from four multi-national data sources such as eICU, HiRID, MIMIC-III, and AUMC, their work aimed to access model transferability across sites internationally. The prediction model reported an AUC of 0.846 on internal cohort validation and 0.761 AUC while validating externally across sites, raising 1.4 false alerts per true alerts. Adams et al.^[^^25^^]^ examined how the range of patient outcomes relates to prompt provider assessment using the Targeted Real-time Early Warning System (TREWS). They observed that their early warning system can identify and prioritize sepsis patients early and thereby improve their prognosis. Benefited from early treatment within 3 h of the alert, after the sepsis alert was confirmed by a provider, the TREWS model demonstrated decrease in organ failure, length of stay, and mortality in hospitalized patients. Wong et al.^[^^26^^]^ observed a substantial drop in test characteristics of the Epic Sepsis Model (ESM), along with an unacceptably high rate of false positives.

For the development of effective risk prediction models, it is crucial that how a problem has been framed and the prediction rules can be improved by the optimal selection of prediction and observation windows. Lauritsen et al.^[^^27^^]^ suggested that for successful risk prediction, temporal framing structures are critical and affect model performance and learning. Applying five ML algorithms on four implemented framing structures, they emphasized on the fundamental need of the concept of framing to build future ML risk prediction models. Goh et al.^[^^28^^]^ introduced an important advancement in EDS through the development and validation of the Sepsis Early Risk Assessment (SERA) algorithm. Utilizing structured data and unstructured clinical notes, the SERA algorithm demonstrates remarkable performance, achieving a high predictive accuracy of 0.94 AUC, with sensitivity and specificity rates of 0.87 each, 12 h before sepsis onset. Comparative analysis against human predictions highlights SERA's potential to significantly enhance sepsis detection by up to 32%, while concurrently reducing false positives by up to 17%. Notably, the algorithm's efficacy surpasses traditional clinical measures, particularly evident in the early warning period 12–48 h before sepsis onset.

### Computational approaches for classification of sepsis levels for MPS

To identify those ICU patients who are at a high risk of deterioration, an interpretable risk stratification predictive model based on Extreme Gradient Boosting (XGB) algorithm is proposed for better MPS, severity assessment, and patient management.^[^^29^^]^ Based on shapley additive explanations (SHAP) for feature identification and prioritization, their multi-source data-driven model is externally validated for satisfactory generalizability and robustness. Bai et al.^[^^30^^]^ analyzed the eICU and MIMIC-IV data, with subsets having sepsis-associated acute respiratory distress syndrome (ARDS). They revealed varying mortality rates and ICU stays and those with sepsis-associated ARDS, with differences observed based on the source of infection. Among these, AdaBoost (DT) demonstrated the highest performance with an area under the receiver operating characteristic curve (AUROC) of 0.895, indicating strong predictive ability. Other algorithms were also evaluated, including RF (AUC=0.874), GB (AUC=0.882), and SVM (AUC=0.834). Even though these algorithms showed competitive performance, they fell slightly short of AdaBoost. Subgroup clustering based on key clinical predictors identified three phenotypes with distinct clinical characteristics and outcomes. These phenotypes exhibited differences in mortality rates and ICU stays across the eICU and MIMIC-IV cohorts. Additionally, the therapeutic effects of different positive end-expiratory pressure levels varied among the phenotypes, with high positive end-expiratory pressure levels associated with higher mortality in clusters 0 and 1 but lower mortality in cluster 2.

### Tailor-made recommender systems for treatment planning

**Figure 2:**
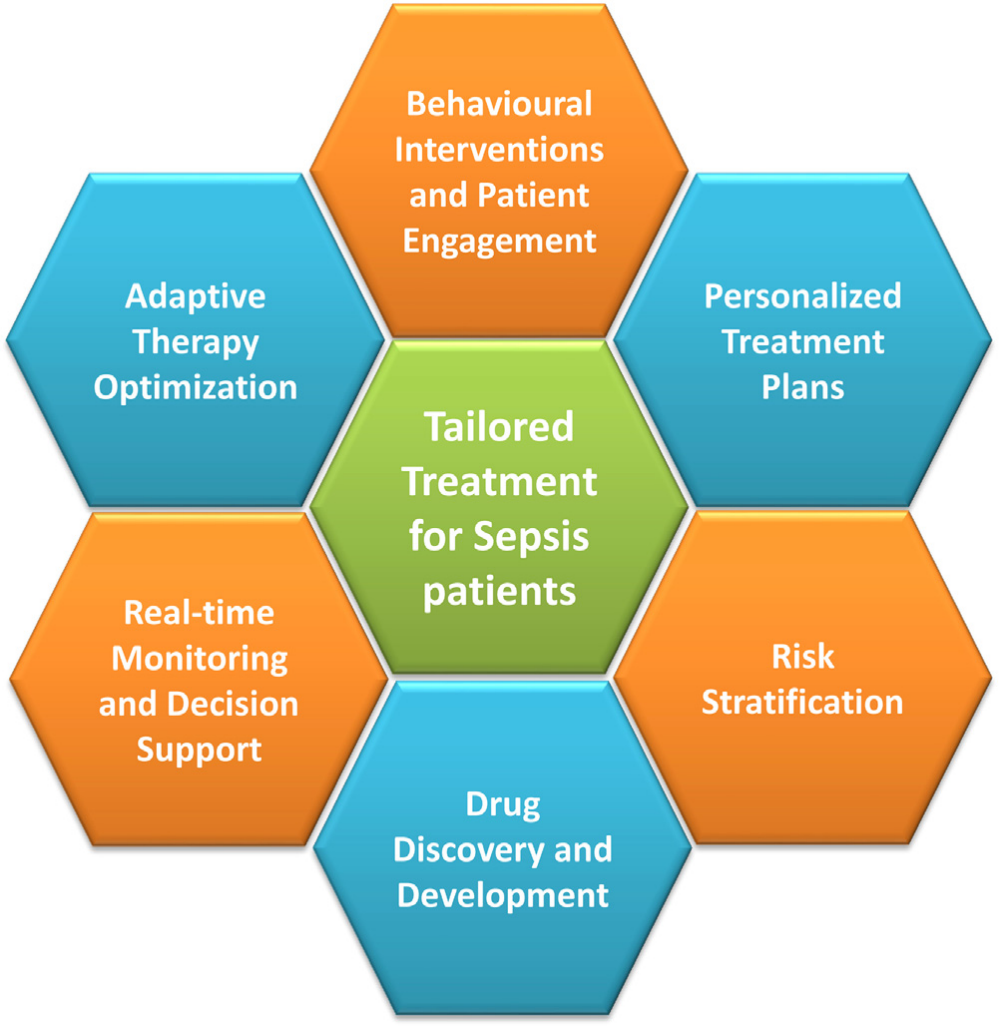
Possible avenues for tailor-made sepsis therapy.

Final treatment plans can be done using tailor-made recommender systems, which focus on clinical and time series data of individual patient. A 6-way approach for customized treatment plan of a patient has been proposed in Figure 2. Use of wearable devices generates data in a time series manner, which can be used by RNNs for customizing treatment plan based on individual patient history. ML models can dynamically adjust treatment parameters based on real-time patient data and feedback, optimizing therapy delivery and dosage according to individual patient responses, disease progression, and evolving clinical circumstances. By examining patterns in patient behavior, preferences, and social factors influencing health, ML can create customized behavioral interventions and support plans.

### Benefits of learning models

Since the conventional models fall short of accurate risk stratification of the disease, computational models based on ML have gained global attention for EDS and MPS. Use of DL models can help in using tabular data with limited sample size such as those <10,000 patients. Autoencoders or deep neural networks (DNNs) can be employed to learn hierarchical representations of the tabular data. Transfer learning can be used in pre-trained models on larger datasets. Tabular data techniques such as adding noise, perturbing feature values, or generating synthetic samples based on the existing data distribution are implemented. Since diagnosis and treatment of sepsis generate a time series of sequential data, reinforcement learning models can be used to analyze data. Ensemble learning models that train multiple DL models with different architectures can reduce variance and enhance the stability of the predictions.

## Data Collection and Popular Data Sources

**Figure 3:**
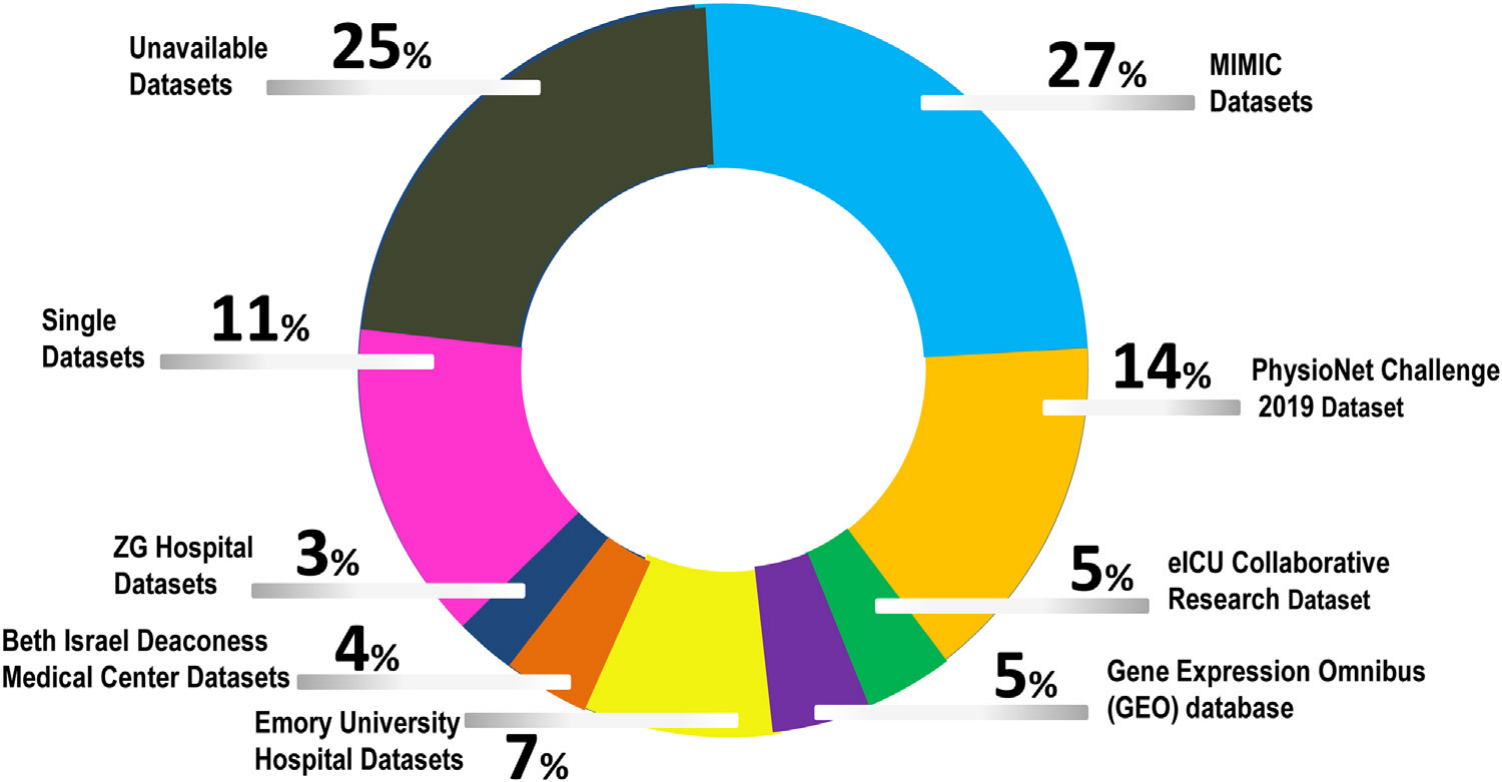
Data share for popularly available data sources. GEO: Gene expression omnibus; ICU: Intensive care unit; ZG: Zigong.

MIMIC-III and MIMIC-IV were employed by major section of researchers for model development and validation.^[^^7^^,^^14^^,^^20^^,^^22^^,^[31], [32], [33], [34], [35], [36], [37], [38], [39]^]^ PhysioNet challenge 2019 dataset is the next popular database used.^[^^8^^,^^15^^,^^16^^,^^20^^,^[40], [41], [42], [43]^]^ The eICU collaborative research database was also utilized by.^[^[33], [34], [35]^]^ Usage of Emory University Hospital dataset and Zigong (ZG) Fourth People's Hospital database, China, was reported in.^[^^33^^,^^37^^,^^41^^,^^44^^]^ A few publicly available discrete datasets were employed by^[^^4^^,^^9^^,^^27^^,^^40^^,^^44^^]^ along with a good number of restricted or unavailable datasets.^[^^22^^,^^24^^,^^25^^,^^28^^,^^30^^,^^31^^,^^34^^,^^36^^,^^38^^,^^41^^,^[45], [46], [47]^]^ As per percentage of data contribution, Figure 3 presents the data share.

## ML for EDS

LR was investigated as one of the four classification algorithms and reported a prediction accuracy of 0.69%.^[^^15^^]^ Proposing “Mixed Filling” for accurate EDS onset up to 6 h before the medical diagnosis, Shankar et al.^[^^16^^]^ investigated the LR classifier with accuracy of 71% and AUROC of 0.75. To calculate the likelihood of developing sepsis within the next 4 h during ICU stay, Bloch et al.^[^^14^^]^ investigated LR as one of the five classifiers on the medical recordings from 8 h. They reported an average performance with AUROC of 0.6541 with split data for the first 36 h and 0.6548 for the first 24 h. Incorporating 13 clinical features, a prediction monogram was developed to effectively predict the ARDS risk in ICU sepsis patients, with an AUROC of 0.811 in the training cohort and 0.812 in the testing set.^[^^48^^]^ Delahanty et al.^[^^49^^]^ employed GB for feature selection introducing the risk of sepsis (RoS) score that provided accurate outcomes, surpassing the performance of screening tools such as SOFA, qSOFA, and SIRS. GB was investigated alongside RF, LR, and DT as classifiers, reporting intermediate performance with 83% accuracy and AUROC of 0.90.^[^^15^^]^ Camacho-Cogollo et al.^[^^42^^]^ designed the robust testing outcomes achieved by XGB, with an 87% accuracy and an AUROC of 0.918 with 24-h data for a prediction time of 1 h. Zhang et al.^[^^39^^]^ harnessed clinical information to construct an ensemble model combining XGB with RF, SVM, and NN for EDS-associated acute kidney injury (S-AKI) onset. Lauritsen et al.^[^^45^^]^ observed that the XGB classifier reported a recall of 0.9934 and accuracy of 93.35% on the “mixed fill” dataset. With a 10-fold cross-validation and hyperparameter tuning, the proposed ensemble model is compared with the individual classifiers including the XGB model, resulting in 96% accuracy and 0.96 AUC.^[^^50^^]^

For accurate EDS onset, RF and LR classifiers were utilized, of which RF was the best, achieving an AUROC of 0.696.^[^^32^^]^ Srimedha et al.^[^^15^^]^ mentioned that the RF model reports an accuracy of 99.01% and AUROC of 99.99%, with only 24 h. Shankar et al.^[^^16^^]^ investigated the RF classifier reporting 97.95% accuracy and 0.9967 AUROC. Singh et al.^[^^50^^]^ proposed an ensemble model of RF, SVM, LR, Naive Bayes (NB), and XGB with 10-fold cross-validation and hyperparameter tuning and reported 96% accuracy. Camacho-Cogollo et al.^[^^42^^]^ reported that the SVM classifier model reveals a high recall. In 1 h prediction time, the ensemble models of SVM with RF and artificial neural network (ANN) reported an accuracy of 84.8% and 78.4%, respectively. Using bedside monitor data, Bloch et al.^[^^14^^]^ aimed to accurately predict a patient's likelihood to develop sepsis within next 4 h of ICU stay. Among the five classifiers, SVM provided highest AUC of 88.38% and accurate onset prediction. Using the decision-tree-based SVM classifiers, the physiochemical prediction model exhibited 68% accuracy in the testing phase.^[^^40^^]^ Comparing the ensemble model of RF, SVM, LR, NB, and XGB with the individual NB model, Singh et al.^[^^50^^]^ reported the performance of the NB classifier with 74% accuracy and 0.74 AUC. Merve et al.^[^^41^^]^ utilized a long short term memory (LSTM) network to successfully forecast sepsis in ICU-admitted patients up to 12 h in advance with 89% accuracy and an AUROC of 94%. The sequential convolutional neural network (CNN)-LSTM model achieved an AUROC of 0.856 when evaluated 3 h before sepsis onset.^[^^41^^]^ For EDS up to 6 h before clinical diagnosis, the LSTM classifier exhibited an average performance with 80.64% accuracy and 0.8190 AUROC.^[^^16^^]^ Al-Mualemi and Lu^[^^40^^]^ used an intelligent LSTM-RNN classifier to validate their DL-based sepsis estimation framework, resulting in a 91.1% testing accuracy. Inger et al.^[^^33^^]^ designed the NAVOY sepsis algorithm using CNN, for prediction of EDS up to 3 h. Al-Mualemi and Lu^[^^40^^]^ developed an intelligent early sepsis estimation framework with LSTM along with the rectified linear unit (ReLU) activation function, showing an accuracy of 93.84% for the training phase and 93.18% for the testing phase. Combining NN with SVM, RF, and XGB, an accurate ensemble model for early prediction of S-AKI was designed, resulting with AUROC values 0.774–0.788 and 0.756–0.813, respectively, 48–12 h before the onset of acute kidney injury (AKI).^[^^39^^]^ Nesaragi et al.^[^^10^^]^ developed an XAI model for EDS (xMLEPS) with clinical interpretability. Chen et al.^[^^4^^]^ assessed a total of 142 features using an ML model and attained a utility score of 0.4274 and an accuracy of 84.88%.

## ML for MPS

Chicco and Jurman^[^^34^^]^ utilized LR to forecast the survival probability based on three features (sex, age, and septic episode number), achieving sensitivity of 0.805 and precision-recall area under the curve (PR-AUC) of 0.941 in the primary cohort, while in the study cohort the sensitivity score is of 0.764 and PR-AUC of 0.826. Hou et al.^[^^51^^]^ employed LR for 30-day mortality prediction, identified through backward step-wise analysis, the chi-squared test, reporting a discriminatory power with an AUC of 0.819. Li et al.^[^^37^^]^ noted LR's lowest AUC of 0.876 found in 5-fold cross-validation in predicting in-hospital mortality. Chicco and Jurman^[^^34^^]^ used GB in survival prediction, achieving AUROC close to 0.7 and highest PR-AUC was 0.966 in the primary cohort. They reported superior performance of the gradient boost decision tree (GBDT) model compared with other methods, achieving an AUC-ROC of 0.992. XGB showed significant predictive value in their 30-day MPS with an AUC of 0.857.^[^^51^^]^ Sankaranarayanan et al.^[^^44^^]^ deployed a DNN model and evaluated its performance with fundamental classifiers including XGB. The DNN model exhibited 98.54% accuracy and 0.98 F1-score in comparison to the XGB classifier with 95.06% accuracy and 0.95 F1-score. van Doorn et al.^[^^13^^]^ emphasized its role in predicting mortality for ED patients, achieving an AUC of 0.82.^[^^23^^]^ In predicting in-hospital mortality for neonates, a better performance of a DNN-based multivariate regression model (95.64% accuracy and 0.77 F1-score) compared with XGB reflecting 81.21% accuracy and 0.39 F1-score was observed.^[^^36^^]^ Kwon and Baek^[^^43^^]^ utilized RF for 3-day mortality prediction. Li et al.^[^^37^^]^ reported a moderate RF performance with an AUROC of 0.98 in predicting ICU MPS. Hsu et al.^[^^36^^]^ highlighted its comparable prediction capabilities with DNN models in neonatal ICU mortality prediction with the RF model achieving 94.24% accuracy and F1-score of 0.69. Jiang et al.^[^^9^^]^ combined ICU readmission and sepsis mortality data, using SHAP to explain feature impact, with an AUC of 0.732 in the sepsis group and 0.83 in the non-sepsis group. Hu et al.^[^^8^^]^ enhanced RF interpretability for ICU MPS, achieving 84.64% accuracy and AUC of 0.81 with SHAP and local interpretable model-agnostic explanations. Real-time personalized treatment recommendations for sepsis treatment using deep reinforcement learning (DRL) have shown promising results. Wu et al.^[^^52^^]^ proposed a weighted dueling double deep Q-Network with embedded human expertise (WD3QNE) achieving survival rates of 97.81% in the MIMIC-III dataset.

## Model Analysis

It becomes imperative to discuss the factors on which the selection of ML models is mostly dependent. Transformers and ensemble models are useful, although little investigation has been done till date. Transformer models are particularly useful for sequential data processing, attention, and pre-trained representations. Sequential data processing can be valuable for analyzing temporal data such as patient vital signs, laboratory values, and clinical notes over time. The attention model finds acceptance for capturing long-range dependencies and identifying important features for predicting sepsis. This helps in identifying patterns and indicators of EDS. Pre-trained representations can be fine-tuned on datasets to improve performance and adaptability. Advanced ensemble models reduce over-fitting.

### Model selection

Typically, five approaches are there in using clinical processes. The use of these parameters and subsequent variables for the input feature vectors of ML models are shown in Table 1. Vital signs serve as important indicators of systemic inflammation and potential sepsis, and those signs are tachycardia, tachypnea, hypotension, and fever. Laboratory tests generate complete blood count (CBC) count. The elevated CBC count is an indicator for ongoing infection. Other important markers are C-reactive protein (CRP) and Procalcitonin (PCT). High levels of lactate levels indicate tissue hypoperfusion. There are further coagulation parameters, such as prothrombin time (PT), activated partial thromboplastin time (APTT) and disseminated intravascular coagulation (DIC), which also serve as important markers. Clinical history forms a major marker for immune status, chronic medical conditions, presence of infection, and recent surgery or invasive procedures. Another important clinical parameter is the clinical assessment, which generates data for hypoperfusion signs, altered mental status, and organ dysfunction. Microbiological and imaging findings help in providing the radiographic evidence of infection, which provide information of a specific infectious source.

**Table 1:** Clinical variables commonly integrated into well-performing models.

Clinical parameters	Analysable parameters for ML models
Vital signs	Temperature Heart rate Respiratory rate Blood pressure Oxygen saturation
Laboratory values	WBC Lactate levels CRP PCT BUN Creatinine Platelet count Coagulation parameters (e.g., PT, INR, APTT)
Clinical scores and indices	SOFA qSOFA APACHE II NEWS PEWS
Clinical history and risk factors	Source of infection (e.g., pneumonia, urinary tract infection, and intra-abdominal infection) Immunocompromised status Recent surgery or invasive procedures Presence of chronic medical conditions (e.g., diabetes, COPD, and heart failure)
Clinical assessment findings	Altered mental status Signs of hypoperfusion (e.g., mottled skin and oliguria) Evidence of organ dysfunction (e.g., renal failure and respiratory distress)
Microbiological and imaging findings	Positive blood cultures Radiographic evidence of infection (e.g., infiltrates on chest X-ray) Identification of specific pathogens (e.g., bacteria and viruses)
Temporal patterns and trends	Changes in vital signs and laboratory values over time Time since onset of symptoms or hospital admission
Demographic information	Age Gender Ethnicity Admission type (e.g., elective vs. emergency)
Healthcare utilization data	Length of stay in the hospital or ICU Previous hospitalizations or healthcare encounters Use of antibiotics or other medications

APACHE II: Acute physiology and chronic health evaluation II; APTT: Activated partial thromboplastin time; BUN: Blood urea nitrogen; CRP: C-reactive protein; COPD: Chronic Obstructive Pulmonary Disease; ICU: Intensive care unit; INR: International normalized ratio; ML: Machine learning; NEWS: National early warning score; PCT: Procalcitonin; PEWS: Pediatric early warning score; PT: Prothrombin time; qSOFA: Quick-sequential organ failure assessment; SOFA: sequential (sepsis-related) organ failure assessment; WBC: White blood cell.

### Model validation and interpretation

Data and feature engineering is the first step in generating valid data for input to the models. Thereafter, splitting of the dataset into training, as well as validation and test sets, forms one of the basic ways of validation, which can be judged using cross-validation techniques. Validating metrics such as accuracy, precision, recall, F1-score, receiver operator characteristic curve, and AUC further help in model validation, apart from resampling and cross-domain validations. XAI aids in gaining trust on the results obtained from the ML models. There are multiple XAI approaches till date.^[^^7^^]^ For interpreting the ML models used for sepsis, till date, local interpretable model-agnostic explanations, SHAP, and descriptive ML explanations (DALEX) have mostly been used.^[^^29^^,^^44^^]^

### Model comparison

Analyzing 16 potential studies on EDS in emergency departments or ICU patients, we observed that six of the studies used data from the MIMIC-III and IV datasets. Four studies developed their models based on the PhysioNet challenge 2019 database.^[^^15^^,^^16^^,^^33^^,^^40^^]^ EDS was done using subsets of a comprehensive set of the following models – RF, LR, GB, DT, SVM, XGB, CNN, K nearest neighbor (KNN), ANN, NB, generative adversarial network, LSTM, LSTM-RNN, adaptive convolutional neural network, light gradient boosting machine, and NN,^[^^15^^,^^16^^,^^32^^,^^33^^,^[38], [39], [40]^,^^42^^,^^48^^]^ whereas MPS was done using RF, LR, KNN, SVM, GBDT, GB, NB, and XGB on the MIMIC and PhysioNet challenge data. The subsets from the ML models were also used on data from the Israel Rabin Medical Center, ED at the Maastricht University Medical Center, Skaraborg Hospital, Four Danish municipalities’ data, data from a tertiary level neonatal intensive care unit in Taiwan, Shanghai Children's Medical Center from 2010 to 2017, and four hospitals of Korea.^[^^13^^,^^14^^,^^36^^,^^37^^,^^43^^,^^45^^,^^53^^]^ Al-Mualemi and Lu^[^^40^^]^ estimated the missing values by capturing temporal dependencies through adversarial NN, while Persson et al.^[^^33^^]^ and Mohammed et al.^[^^32^^]^ used forward-filling for missing data processing. Shankar et al.^[^^16^^]^ proposed “Mixed Filling” as a new imputation algorithm with K-fold cross-validation. Camacho-Cogollo et al.^[^^42^^]^ imputed the missing data with KNN imputation, while El-Rashidy et al.^[^^38^^]^ used the expectation-maximization algorithm. Nearly all the ML models in these studies have reported AUROC scores more than 0.8, with a major portion recorded even over 0.9. In comparison to the traditional predictive tools with the values around 0.7, the reported scores are significantly higher.

We investigated 10 studies for MPS in emergency departments or ICU patients and observed that most of the studies used different datasets – MIMIC – III v1.4 & IV datasets,^[^^8^^,^^9^^,^^37^^,^^54^^]^ data collected from child hospitals,^[^^44^^]^ two data sources of the Norwegian patient registry and statistics and South Korean patient data.^[^^34^^]^ The situation of imbalance in the datasets was minimized with the help of Randomly Over Sampling Examples (ROSE) oversampling.^[^^34^^]^ While Hou et al.^[^^51^^]^ used maximum, minimum, and mean values, Li et al.^[^^37^^]^ opted for means in each group for the missing values. Considering the missingness of data to be missing at random, the multivariate imputation technique was followed.^[^^8^^,^^9^^]^ The confusion matrix evaluation scores are used as standards to evaluate the model performance in MPS. AUROC, PR-AUC, F1-score and accuracy are used as the prediction scores. Since they emphasize on the true positive rates attained by each model, the PR-AUC scores are crucial.

## Challenges and Limitations

### Major challenges for EDS and MPS

The major challenges in the use of computational models are the availability and quality of data, including missing values, noisy measurements, and inconsistent documentation.^[^^42^^,^^48^^]^ The ratio of sepsis vs. non-sepsis cases is relatively high, resulting in imbalanced datasets.^[^^10^^]^ Addressing this issue requires appropriate data sampling techniques to balance the dataset. ICU patients present complex and diverse clinical characteristics, including comorbidities, organ dysfunctions, and diverse treatment interventions.^[^^46^^,^^55^^]^ Ensuring that the developed models generalize well to different patient populations and healthcare settings is crucial.^[^^56^^]^ The level of dysfunction with respect to sepsis III can be sub-divided into the following multiple organ dysfunctions.^[^^57^^]^

Sepsis is often linked with lung dysfunction, commonly referred to as ARDS or acute lung injury. Cardiovascular dysfunction occurs during the early stages of sepsis. It has significantly increased the mortality rate to 70%–90%. Liver dysfunction is based on the increase in bilirubin concentration >2 mg/dL and the occurrence of coagulation disorders with international normalized ratio >1.5.

Kidney dysfunction stands out as a major contributor to AKI and is correlated with elevated mortality rates and manifests in 1–35% of hospitalized patients. Severe sepsis can cause hyperactive or hypoactive delirium, seizures, and cerebrovascular events due to Central Nervous System (CNS) dysfunction. In cases of severe sepsis, the blood coagulation system undergoes widespread activation, leading to the consumption of multiple clotting factors and resulting in DIC.Sepsis may decrease microcirculation in the gastrointestinal tract in patients and increase the gastrointestinal bleeding risk of stress-related mucosal disease.

### Data limitations

Variability in symptoms, clinical presentations, and laboratory results can make it difficult to establish clear diagnostic criteria or predict outcomes accurately due to data variability. Inconsistent or missing information about clinical parameters, comorbidities, and patient history can hinder the analysis. Noise in healthcare data can arise from errors in data entry, sensor measurements, or variability in clinical practices. Healthcare data comes from various sources, including electronic health records (EHRs), laboratory reports, and imaging studies. These sources may use different formats and standards, making it challenging to integrate and analyze data effectively. Patient data are sensitive, and privacy regulations can limit data sharing and research. These concerns may restrict access for research. Sepsis can evolve rapidly, and the availability of timely data is crucial for accurate diagnosis and prediction. Delays in data entry, retrieval, or analysis can affect the timeliness of interventions and decisions. Sepsis is a relatively rare condition, and this can result in imbalanced datasets. Ensuring that computational models do not discriminate against certain demographic groups or exhibit bias in their predictions is a significant ethical concern. Updates, such as sepsis-3 criteria, can create challenges when working with historical datasets.

### Data generalization challenges

Data generalization involves creating models that can make accurate predictions or classifications on new, unseen data.

There may be a scarcity of data, leading to overfitting or underfitting. When the distribution of the training data differs significantly from the distribution of the new data, the model's performance may deteriorate due to this data distribution pattern.

Feature engineering can hinder a model's ability to generalize across different datasets. Choosing the right hyperparameters for an ML model is crucial for achieving good generalization. Effective cross-validation techniques and proper evaluation metrics are necessary to avoid overly optimistic estimates of a model's performance. In imbalanced class distributions, models can struggle to generalize effectively to the minority class. Dealing with large number of categories can be challenging. Encoding, selecting, and representing categorical data effectively are essential for generalization. Cleaning, normalizing, and transforming the data appropriately are essential for proper generalization. Obtaining accurate and reliable labels for training data can be challenging, leading to the data annotation and labeling issue.

### Model limitations

**Figure 4:**
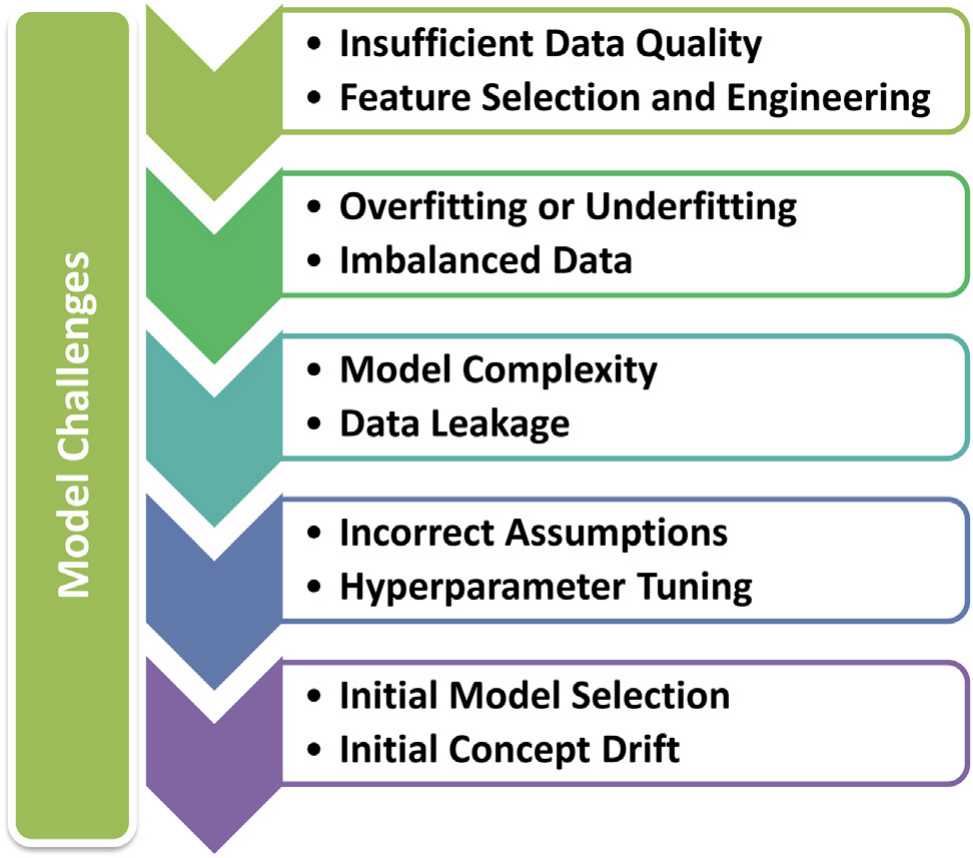
Potential reasons for under-performance of predictive models.

There are certain reasons for under-performance of predictive models as shown in Figure 4. Apart from these, we discuss the other major model challenges: (1) Choosing appropriate ML algorithms and architectures that are well-suited for the specific task. For example, DT, RF, GB, SVM, and NN have different strengths and weaknesses. (2) Challenges exist in designing XAI models. (3) Implementing transfer learning techniques for pre-trained models on related medical tasks. (4) Stacking multiple models, each with different strengths and weaknesses, into an ensemble. Ensemble methods, such as bagging and boosting, can improve model robustness and generalization. (5) Conduct thorough clinical validation and testing of the model using real-world data in a clinical setting. (6) To ensure that model predictions are well-calibrated. Calibration techniques can help fine-tune predictions to be more accurate.

### Ethical and privacy concerns

AI has resulted in raising ethical concerns among the black-box models, to the extent of terming healthcare applications as the high risk factors, in tune with General data protection regulation.^[^^47^^]^ Developing frameworks for assessing algorithmic accountability is necessary. Regular monitoring of the model's performance in a real clinical environment and updating it as new data becomes available, ensuring that de-identified data remain anonymous and cannot be easily re-identified are a technical and ethical challenge. Managing data that have already been used in model development when consent is withdrawn is a challenging, logistical issue. Models must meet regulatory standards before they can be deployed in a clinical setting. Resolving data ownership disputes and ensuring data access for research while respecting patient rights is challenging. Knowing who is responsible when a model makes a wrong diagnosis or prediction is challenging, but necessary.

## Observed Results and Inferences

Supplementary Figure S1 presents the performance metrics with respect to the benchmark MIMIC and PhysioNet datasets. The metrics are provided in terms of the F1-score, accuracy and AUC-ROC. It can be observed that the literature has witnessed an F1-score range within 0.2700 and 0.9978. Range for accuracy falls within 0.3370 and 0.9977. The range for AUROC lies within 0.4990 and 0.9999.

## Future Research Directions

Real-time monitoring systems, facilitated by wearable devices and Internet of Things sensors, are poised to enable continuous patient surveillance, enhancing early detection. XAI methods will become increasingly important for transparent decision-making. Longitudinal data analysis, robust validation through clinical trials, and a focus on ethical considerations will shape the future landscape of sepsis prediction.

Wearable devices take center stage, becoming vital instruments for continuous monitoring, by seamlessly blending into patients’ daily lives. The intricate connectivity of the Internet of Things within healthcare settings has become a catalyst for dynamic, real-time data exchange. Clinical workflow has huge prospects in society, in terms of developing real-time decision support systems that seamlessly integrate with EHRs and provide timely alerts to healthcare providers when there is a high RoS. Technology-assisted clinical workflow should explore methods to automate the capture of relevant patient data for input into predictive models. Investigating ways to integrate predictive models with mobile apps and wearable technologies to extend monitoring beyond the hospital setting are an open domain.

Ensemble methods, RNNs, and attention mechanisms can capture complex patterns in multi-modal data. Another uninvestigated avenue is the domain of real-time monitoring and alerts. XAI techniques can help clinicians understand the reasoning behind predictions and facilitate more informed decision-making.

## Conclusion

This study is a comprehensive study on existing computational approaches for EDS and MPS, and documents which ML models have been used. Publicly available data sources have been outlined for further study. This serves amateur researchers in delving into the topic with valid benchmark datasets and serves as a benchmark for initiating research in this domain, and also presents the challenges and limitations with respect to using computation in this field, which serves as a firsthand knowledge of the research gaps. Finally, we present the future research possibilities for EDS and MPS.

## Appendix Supplementary materials

Image, application 1
