# Supplementary material for: Investigating computational models for diagnosis and prognosis of sepsis based on clinical parameters: Opportunities, challenges, and future research directions
Source: J Intensive Med. 2024 Jul 10;4(4):468–77. doi: 10.1016/j.jointm.2024.04.006 (PMC11411432; doi:10.1016/j.jointm.2024.04.006)
Supplement: Supplementary file 1 [file mmc1.docx]

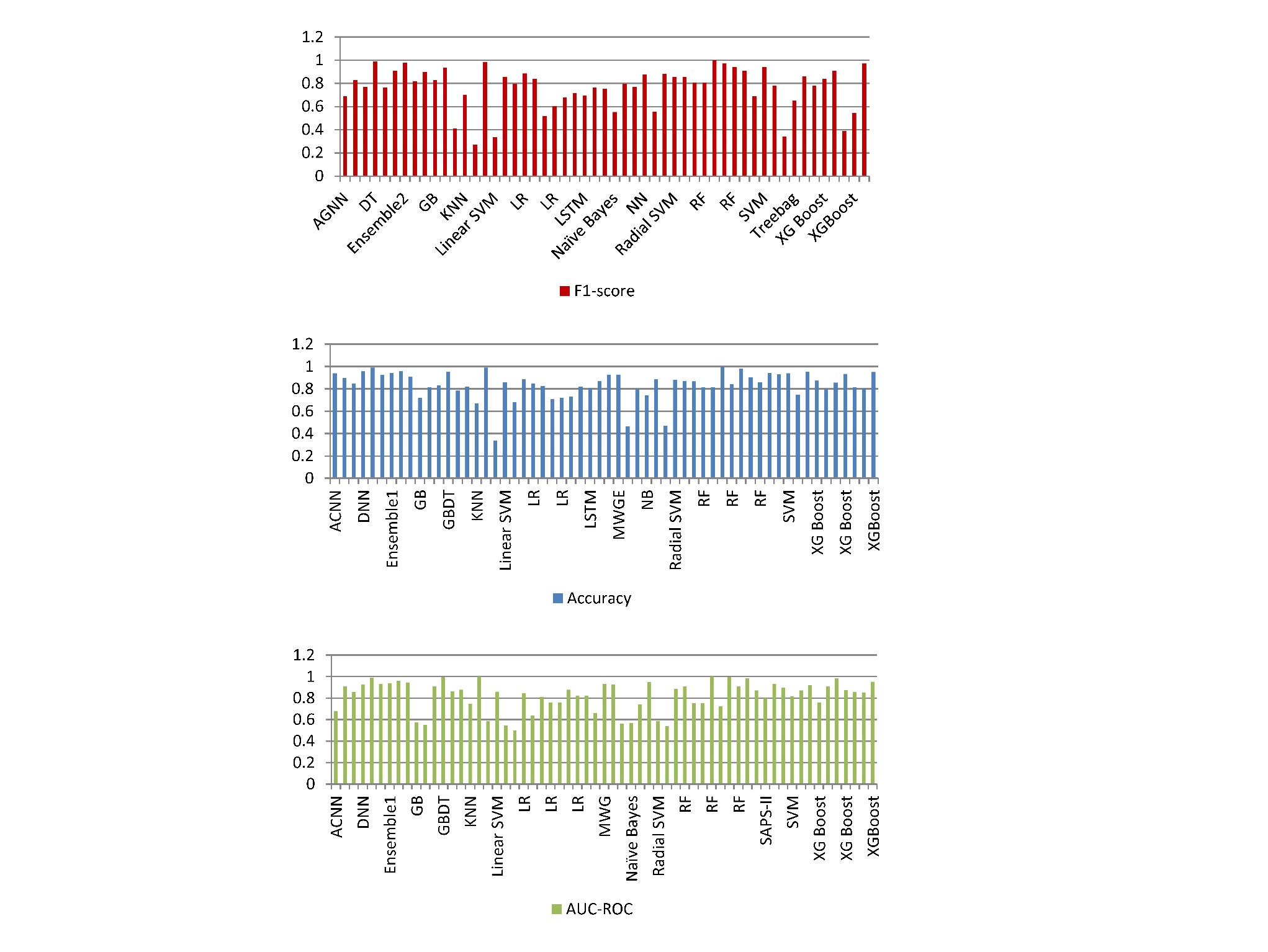


**Figure S1.** Performance metrics with respect to the benchmark MIMIC and PhysioNet datasets on various ML models.

ACNN: Adaptive Convolutional Neural Network; ANN: Artificial neural networks; DT: Decision tree; GAN: Generative adversarial network; GB: Gradient boosting; GBDT: Gradient boost decision tree; KNN: K nearest neighbor; LGBM: Light gradient boosting machine; LR: Logistic regression; LSTM: Long–short- term memory; MLP: Multi-layer perceptron; MWG: Model without graph neural networks; MWGE: Model without graph neural networks and Ensemble Learning; NB: Naive Bayes; NN: Neural network; RF: Random forest; SAPS: Simplified acute physiology score; SVM: Support vector machine; XG Boost: Extreme gradient boosting.
